# The Public Services

**Published:** 1913-09-06

**Authors:** 


### THE PUBLIC SERVICES.

### ROYAL NAVAL MEDICAL SERVICE.

*Qualification.*—1. Every candidate for admission into the Medical Department of the Royal Navy must be not under 21 nor over 28 years of age on the day of the commencement of the competitive examination. He must produce an extract from the register of the date of his birth, or, in default, a declaration made before a magistrate stating the date of birth.

2. He must declare: (1) His age and date and place of birth; (2) that he is of pure European descent, and the son of either natural born British subjects or of parents naturalised in the United Kingdom; (3) that he labours under no mental or constitutional disease or weakness, nor any other imperfection or disability which may interfere with the most efficient discharge of the duties of a medical officer in any climate; (4) that he is ready to engage for general service at home or abroad if required; (5) whether he holds, or has held, any commission or appointment in the Public Services; (6) that he is registered under the Medical Act, giving the date of his registration as a medical student or of his beginning professional study; (7) whether he has been previously examined for entry into the Naval Service, and, if so, when.

3. The certificates of registration and birth must accompany the declaration, which is to be filled up and returned as soon as possible, addressed to the Director-General, Medical Department, Admiralty, S.W., before the candidate can be allowed to compete for an existing vacancy.

4. The candidate will be examined as to his physical fitness by a Board of Naval Medical Officers.

5. Candidates who have served in the Officers' Training Corps will be credited with additional marks at the entrance examination in the following proportion: Those in possession of Certificate A, 1 per cent.; those in possession of Certificates A and B, 2 per cent. of the maximum number of marks allotted.

*Examination.*—1. Entrance examination held twice yearly in London in the following subjects: (*a*) Medicine, including medical pathology and therapeutics; (*b*) surgery, including surgical pathology and clinical surgery. The examination will be partly written and partly practical, marks being allotted under the following scheme:—

| Medicine. | |
|---|---|
| Paper ... ... ... ... ... ... | 400 |
| Clinical ... ... ... ... ... ... | 400 |
| Oral ... ... ... ... ... ... | 400 |
| | 1,200 |

| Surgery. | |
|---|---|
| Paper ... ... ... ... ... ... | 400 |
| Clinical ... ... ... ... ... ... | 400 |
| Oral ... ... ... ... ... ... | 400 |
| | 1,200 |

No candidate will be considered eligible unless he obtains 50 per cent. of the marks in each subject.

Successful candidates will be given appointments as acting surgeons in the Royal Navy, and will then enter upon a six months' course of training.

The first two months will be passed at the Naval Medical School, Greenwich. Here they will receive instruction in tropical medicine, pathology, and hygiene.

The remaning four months will be passed at Haslar, and will be devoted to the study of naval hygiene, recruiting, physical training, diving and submarine work, radiography, anæsthetics, dentistry, etc.

At the conclusion of the Greenwich and Haslar courses examinations will be held, and the marks gained in these, added to those gained at the entrance examination, will determine the seniority of the commissions granted to the successful candidates as Surgeons, R.N.

2. A further examination is held for the rank of staff-surgeon at Greenwich and London twice yearly, and follows on the course of instruction described on the following page.

*Post Graduate Courses.*—1. Prior to presenting themselves for the examination for qualification for the rank of staff-surgeon officers are provided with a complete course of instruction of five months' duration at the Royal Naval Medical School, Greenwich, the "Dreadnought" Hospital, Greenwich, and other civil hospitals in London, as may be arranged from time to time.

This course of instruction comprises: (*a*) Clinical medicine and clinical surgery; (*b*) operative surgery; (*c*) anæsthetics (practical); (*d*) ophthalmology; (*e*) clinical pathology; (*f*) hygiene; (*g*) throat, nose, and ear diseases; (*h*) skiagraphy.

2. A course of three months' duration for officers of not less than fourteen years' seniority is arranged for twice yearly, and is directed to affording them opportunities of acquiring special knowledge.

3. In addition, courses of instruction of about six weeks duration in clinical work and hospital administration are arranged for at each of the home ports for the benefit of those officers who may be in the ports at the time.

There are no formal examinations at the end of these courses, but they have a certain bearing on the progress of officers, as reports concerning them are transmitted to headquarters.

*Distinctions.*—(*a*) Prizes given at end of acting surgeons' course of instruction, viz.: A gold and silver medal and three surgical pocket-cases; (*b*) Gilbert Blane medal. (This gold medal is awarded annually to the medical officer who obtains the highest aggregate marks at the half-yearly examinations for promotion to staff-surgeon; (*c*) Chadwick Naval Prize (£100, once in five years, for distinguished work in naval hygiene); (*d*) King's honorary physicians and surgeons.

*Promotions.*—Promotion to staff-surgeons and fleet-surgeons takes place at the end of eight and sixteen years respectively—provided the qualifying examination for staff-surgeon has been passed. In addition, special promotion will be made at their Lordships' discretion as a reward for distinguished service or conspicuous professional merit.

Accelerated promotion will be granted to officers who at the qualifying examination for staff-surgeon obtain at least 75 per cent. of the total marks.

Promotion to the ranks of deputy surgeon-general and surgeon-general are made by selection from the ranks of fleet-surgeons and deputy surgeons-general respectively.

*Withdrawals.*—Officers will be allowed to withdraw, at the discretion of their Lordships, with gratuities on the following scale: After four years' service, £500; after eight years' service, £1,000; after twelve years' service, £1,500; and after sixteen years' service, £2,250.

For retired pay and widows' pensions see the Regulations for the Naval Medical Service.

Officers joining the Service at the present time can feel assured of practically continuous employment.

### THE ROYAL ARMY MEDICAL CORPS.

A candidate for a commission in the Royal Army Medical Corps must be 21 years and not over 28 years of age at the date of the commencement of the Entrance examination. He must be registered under the Medical Acts in force in the United Kingdom. Before being permitted to proceed to the Entrance examination, the candidate is required to attend at the War Office about two days before the commencement for the purpose of being interviewed and undergoing physical examination. The Entrance examination is held twice a year in London—January and July—a fee of £1 being charged for admission thereto. It lasts about three days. The examination is of a clinical, written, practical, and oral character. It consists of: A commentary on a case in medicine; a commentary on a case in surgery; examination and report on a clinical medical case; examination and report on a clinical surgical case; orals in clinical medicine, clinical surgery and in medical and in surgical pathology; also surgical operations and bandaging (including surgical instruments and appliances). A candidate who is successful at the Entrance examination is appointed a lieutenant, (on probation) and is then required to attend at the Royal Army Medical College, London, and afterwards at the Royal Army Medical Corps School of Instruction, Aldershot, for the purpose of qualifying in the following subjects before his commission can be confirmed:—Royal Army Medical College: Military surgery, tropical medicine, hygiene, pathology, and organisation of military hospitals, and principles governing medical charge of troops. Royal Army Medical Corps School of Instruction: Regimental duties, drill and field training.

### THE INDIAN MEDICAL SERVICE.

Candidates must be natural-born subjects of his Majesty, of European or East Indian descent, between 21 and 28 years of age at the date of the examination, of sound bodily health, and in the opinion of the Secretary of State for India in Council in all respects suitable to hold commissions in the Indian Medical Service. They may be married or unmarried. They must possess, under the Medical Acts in force at the time of their appointment, a registrable qualification to practise both medicine and surgery in Great Britain and Ireland.

The physical fitness of each candidate will be determined by a Board of Medical Officers, who are required to certify that his vision is sufficiently good to enable him to pass the tests laid down by the Regulations. The candidate will be examined by the Examining Board in the following subjects:

(1) medicine, including therapeutics; (2) surgery, including diseases of the eye; (3) applied anatomy and physiology; (4) pathology and bacteriology; (5) midwifery and diseases of women and children; (6) materia medica, pharmacology, and toxicology. The examination in medicine and surgery will be in part practical, and will include operations on the dead body, the application of surgical apparatus, and the examination of medical and surgical patients at the bedside. No syllabus is issued in the subjects of the examination which will be conducted so as to test the general knowledge of the candidate in these subjects. No candidate shall be considered eligible who shall not have obtained at least one-third of the marks obtainable in each of the above subjects and one-half of the aggregate marks for all the subjects.

After passing examination, the successful candidates will be required to attend a course of practical instruction at the Army Medical School and elsewhere, as may be decided, in: (1) hygiene; (2) military and tropical medicine; (3) military surgery; (4) pathology of diseases and injuries incidental to military and tropical service. This course will be of not less than four months' duration, and at its conclusion candidates will be required to pass an examination in the subjects taught therein.

### THE COLONIAL MEDICAL SERVICE.

Full details regarding this service and the advantages it offers to newly qualified men may be obtained on application to the Private Secretary, Colonial Office.

